# Effect of iron administration on the aortic iron content and vascular calcification in phosphorus-loaded chronic kidney disease rats

**DOI:** 10.1186/s12882-023-03426-5

**Published:** 2023-12-15

**Authors:** Masa Nakanishi, Ayako Goto, Takahide Iwasaki, Takeshi Nakanishi, Akihiro Kuma, Masayoshi Nanami, Takahiro Kuragano

**Affiliations:** Division of Kidney, Dialysis and Cardiology, Department of Internal Medicine, Hyogo Medical University, Nishinomiya, 663-8501 Hyogo Japan

**Keywords:** Chronic Kidney Disease, iron, Phosphorus, Vascular calcification, Nrf2, Ferritin H, HIF-1α

## Abstract

**Background:**

Cardiovascular disease (CVD) is a major cause of morbidity and mortality in patients with chronic kidney disease (CKD) and could be related to oxidative stress. Vascular calcification (VC) has been established as a critical risk factor for accelerated CVD. In CKD, phosphorus (Pi), iron (Fe) and Nrf2 are modulators of VC and important agonists and antagonists of oxidative stress. The aim of this study was to determine whether Fe administration, which is commonly used to treat renal anemia, affects aortic Fe overload and VC, and whether Nrf2 and its related genes, ferritin H and HIF-1α, are involved in the development of VC.

**Methods:**

A CKD model was created in rats by administering adenine and simultaneously feeding a high-Pi diet. In addition to control and CKD rats without Fe administration (No-Fe group), Fe was administered orally (PO-Fe group) or intraperitoneally (IP-Fe group) to CKD animals to clarify the effects of Fe administration on the aortic Fe and calcium (Ca) contents and the involvement of Nrf2 and its induced antioxidative proteins, ferritin H and HIF-1α, in VC.

**Results:**

The aortic Fe content increased significantly in the IP-Fe group, which was closely correlated with liver HAMP (hepcidin) expression in all animals. Fe administration had no significant effect on the aortic Ca and Pi contents regardless of the route of Fe administration. The aortic mRNA level of Nrf2 was significantly increased in the IP-Fe group and correlated with serum Pi levels and aortic Fe contents, which could respond to oxidative stress. Notably, the mRNA level of Nrf2 was also significantly correlated with the mRNA levels of ferritin H and HIF-1α. Since we could not measure Nrf2 protein levels in this study, we confirmed the upregulation of HMOX1 and NQO1 mRNA expression in parallel with Nrf2 mRNA.

**Conclusion:**

Parenteral Fe administration increased aortic Fe in parallel with the liver HAMP mRNA level but did not affect VC. Aortic Nrf2 mRNA levels correlated significantly with aortic Fe and serum Pi levels and with aortic mRNA levels of ferritin H and HIF-1α as well as HMOX1 and NQO1.

## Introduction

Cardiovascular disease (CVD) is a major cause of morbidity and mortality in patients with chronic kidney disease (CKD) [1, 2]. Numerous studies strongly suggest that the risk of developing CVD, especially vascular calcification (VC), is overwhelmingly higher in CKD than in non-CKD patients and may be related to oxidative stress caused by the interplay of hyperphosphatemia and dysregulated iron (Fe) metabolism [3–5].

Hyperphosphatemia is evident as CKD progresses into late stages due to inappropriate excretion of phosphorus (Pi) in the urine. Higher Pi concentrations in mitochondria could increase the mitochondrial membrane potential (Δψ), which is known to positively correlate with the production of reactive oxygen species (ROS) in the electron transport chain [6–8].

In CKD, anemia is also an inevitable complication and is related in part to the disruption of normal Fe metabolism that is associated with high hepcidin levels [9]. Fe therapy is very common in the treatment of renal anemia for the prevention of Fe deficiency. Trying to avoid Fe deficiency is likely to induce excess Fe administration. Treatment with Fe preparations necessarily promotes liver-derived hepcidin production [10]. Hepcidin, the master regulator of Fe metabolism encoded by the HAMP gene, interacts with the mammalian cellular iron exporter ferroportin (FPN) and promotes its degradation, which causes intracellular iron sequestration by reducing its efflux. An increase in intracellular Fe leads to a “Fenton-type” reaction, which produces highly reactive hydroxyl radicals and causes severe oxidative stress [11–13]. However, whether hepcidin causes Fe sequestration in aortic tissue in vivo has not been determined. In addition, the role of Fe administration in the pathogenesis of VC has been the focus of several studies with conflicting results, both in vitro and in vivo [12, 14–17], whereas the role of Pi has been firmly established [18, 19].

Oxidative stress can be viewed as a disturbance in the balance between oxidant production and antioxidant defense. An imbalance in favor of prooxidants can lead to the oxidation of several molecules, resulting in tissue injury. In the attenuation of oxidative stress, the involvement of nuclear factor erythroid 2 (NF-E2) p45-related factor 2 (NRF2; encoded by NFE2L2) is presumed to play an important role by upregulating downstream genes related to phase II detoxification enzymes and antioxidant proteins [20, 21]. Indeed, Nrf2 attenuates VC in cultured vascular smooth muscle cells (VSMCs) [20]. However, the effects of Nrf2 on VC remain to be elucidated in vivo, as Nrf2 has been reported to induce the expression of ferritin H, which might suppress VC, as well as Hypoxia inducible factor-1α (HiF-1α), which promotes VC [22–26].

The purpose of the present study was to clarify whether Fe administration promotes the progression of aortic Fe accumulation and affects VC in a rat CKD model with high Pi intake. We also tested the involvement of Nrf2 and its related genes, ferritin H and HIF-1α, in the development of aortic Ca deposition.

## Methods

### Study design and animal care

All protocols were reviewed and approved by the Hyogo Medical University Animal Experiment Committee (No. 17–032). Twenty-four male Sprague‒Dawley rats were purchased from Oriental Yeast Co., Ltd., (Tokyo, Japan) at 10 weeks of age on two separate occasions and conditioned on a standard diet (AIN93M) for one week before the start of each protocol. The rats were housed in cages (two animals in each cage) and allowed free access to food and tap water. The animal room was kept on a 12-h light/dark cycle (7:00 a.m. to 7:00 p.m. lights on, 7:00 p.m. to 7:00 a.m. lights off) at a constant temperature (23 ± 1 °C) and relative humidity of 40–60% throughout the experimental period.

After the conditioning period, the 12 rats were randomly divided into 4 groups: the control group and three CKD groups, specifically, the no iron group (No-Fe group), oral iron group (PO-Fe group) and intraperitoneal iron group (IP-Fe group). Control rats were continuously fed the standard diet for 6 weeks until sacrifice (control group). The other rats were fed a 0.75% adenine-containing Pi-enriched diet (1.20% phosphorus, 1% calcium, 2.4 IU/g vitamin D3) for two weeks. In the following 4 weeks, CKD rats without iron administration were fed the same adenine-containing Pi-enriched diet (No-Fe group). CKD rats with peroral iron were fed a 0.75% adenine-containing Pi-enriched diet supplemented with ferrous sulfate (1000 mg/kg rat chow) (PO-Fe group). CKD rats with intraperitoneal iron were fed a 0.75% adenine-containing Pi-enriched diet and were injected with 40 mg of Fe (saccharated ferric oxide (SFO), FESIN® Intravenous Injection, Nichi-Ikou,Toyama, Japan) intraperitoneally on weeks 2, 3, 4 and 5 (IP-Fe group). After the experimental period (6 weeks), all rats were sacrificed by heart puncture under isoflurane anesthesia (according to the Regulations for Animal Experimentation of Hyogo Medical University). Then, blood samples were collected, and the aorta and liver were dissected for fixation in 10% formalin neutral buffered saline or frozen at -80 °C until later analyses. Studies were conducted in duplicate, the second time with 16 animals, and the results were analyzed together. Induction of CKD using 0.75% adenine for more than 3 weeks, which is considered to yield a high rate of VC, has a high mortality rate, as previously reported, and there were 1–2 missing data points in each group in this study [27].

### Blood tests

Blood levels of creatinine (Crea), urea nitrogen (BUN), total CO_2_ (TCO_2_), hemoglobin (Hb), hematocrit (HTC) and ionized calcium (iCa) were measured by using an iSTAT analyzer3000 (Abbott Japan LLC, Tokyo Japan). The Pi concentration was measured in a commercial laboratory (SRL Co., Tachikawa City, Japan).

### Evaluation of the aortic contents of elements (Ca, Pi, and Fe)

For quantitative evaluation of the aortic contents of Ca, Pi and Fe, stored tissue was weighed and dissolved in concentrated nitric acid. The Ca and Fe contents of the supernatant were determined by the inductively coupled plasma-atomic emission spectroscopy (ICP‒AES) method, and Pi was evaluated by molybdenum blue spectrophotometry at YAGAI KAGAKU Co., Ltd. Sapporo, Japan). These contents were normalized by the tissue dry weight (DW).

### Metallo assay

The hepatic iron content was measured using an iron assay kit according to the manufacturer’s instructions (Metallo assay, Metallogenics, Chiba, Japan). These contents were normalized to the protein content determined by a Pierce™ BCA Protein Assay Kit (Life Technologies Japan, Ltd., Tokyo).

### Tissue iron deposition assay

For liver tissue specimens, sections were cut at a thickness of 10 μm, and staining was performed according to the Berlin blue staining set (Fujifilm Wako Pure Chemical Corporation). We also evaluated the % Fe staining area of liver tissue using ImageJ software (National Institutes of Health, USA) (http://rsb.info.nih.gov/ij/).

### Quantitative real-time PCR

The mRNA expression of Nrf2, Hif-1α, ferritin H, Runt-related transcription factor 2 (RUNX2), Heme Oxygenase1 (HMOX1) and NAD(P)H quinone dehydrogenase 1 (NQO1) in the aorta was measured by real-time polymerase chain reaction (RT‒PCR) analysis. HAMP (hepcidin) expression in the liver was also measured by RT‒PCR analysis. RNA was isolated from cells using TRIzol (Invitrogen, Carlsbad, CA) according to the manufacturer’s protocol. Two micrograms of RNA was reverse transcribed to cDNA with a High-Capacity RNA to cDNA™ Kit (Applied Biosystems, Foster City, CA). RT‒PCR was performed using TB Green II (TaKaRa Bio, Inc., Shiga Japan). Relative mRNA expression was calculated with the 2^−ΔΔCt^ method using β-actin as an internal control. The PCR primer sequences for detecting HAMP, Nrf2, ferritin H, HIF-1α, RUNX2, Hmox1, Nqo1 and β-actin are shown in Table 1. The HIF-1α primers were synthesized by ThermoFisher Japan, and other primers were purchased from TaKaRa Bio, Inc., Shiga, Japan.

**Table 1:** Primer sequences used in the qRT‒PCR analysis

Gene	Forward	Reverse
HAMP	5’-CTGAGCAGCGGTGCCTATCT-3’	5’-GCACTGTCATCAGTCTTGCTTTC-3’
Nrf2	5’- GCTGCCATTAGTCAGTCGCTCTC-3’	5’-ACCGTGCCTTCAGTGTGCTTC-3’
Ferritin H	5’- ACCAGCGAGGTGGACGAATC-3’	5’-ATTCAGGTAATGCGTCTCAATGAAG-3’
HIF-1α	5’- GCTACAAGAAACCGCCTA-3’	5’-GTTCTTCTGGCTCATAACCC-3’
RUNX2	5’- CACAGGGTGACTCCCGTTACAA-3’	5’- TGTGACCCAGTGCAAATGAAGA-3’
Hmox1	5’-ATTTGTCCGAGGCCTTGAA-3’	5’-CCAGGGCCGTATAGATATGGTA-3’
Nqo1	5’-TGAGCCCGGATATTGTAGCTGA-3’	5’-GCATACGTGTAGGCGAATCCTG-3’
β-actin	5’- GGAGATTACTGCCCTGGCTCCTA-3’	5’-GACTCATCGTACTCCTGCTTGCTG-3’

### Statistical analyses

Statistical analyses were performed using R version 3.6.1 (R foundation for statistical computing, Vienna, Australia). Data are presented as the mean ± SD. Differences among groups were analyzed by an unpaired t test for two groups and one-way ANOVA followed by the Tukey‒Kramer test for more than three groups. A two-tailed p value of < 0.05 was considered statistically significant. Univariable linear regression analyses were performed to determine correlations among parameters.

## Results

### Blood biochemical parameters

Table 2 shows the results of the biochemical analyses and body weight at 6 weeks in adenine-induced CKD rats fed a Pi-enriched diet. Crea, BUN, and Pi were higher in all CKD groups (No-Fe, PO-Fe, and IP-Fe groups) than the levels in the control group. The TCO_2_ and iCa levels were significantly decreased in all CKD groups. The iCa level was significantly lower in the IP-Fe group than in the No-Fe and PO-Fe groups. Hb and hematocrit levels were lower in all CKD groups than in the control group.

**Table 2:** Effect of iron (Fe) administration on blood tests and body weight in adenine-treated rats fed a high-phosphorus diet

	Control	No-Fe	PO-Fe	IP-Fe
Na mmol/l	134.8 ± 1.38	136.5 ± 2.5	133.3 ± 3.38	129.8 ± 3.4
K mmol/l	5.7 ± 0.26	3.2 ± 0.2 ^*1^	3.33 ± 0.07 ^*1^	3.10 ± 0.15 ^*1^
Cl mmol/l	98.5 ± 0.65	107 ± 3	104 ± 0.58	101.5 ± 3.93
iCa mmol/l	1.40 ± 0.06	1.10 ± 0.04	1.07 ± 0.06 ^*1^	0.67 ± 0.07 ^*1,*2,*3^
TCO_2_ mmol/l	29.3 ± 0.63	15.0 ± 1.0 ^*1^	17.3 ± 0.33 ^*1^	12.5 ± 1.04 ^*1,*3^
Glu mg/dL	219.5 ± 9.68	132.0 ± 2.0 ^*1^	144.0 ± 2.5 ^*1^	130.5 ± 10.2 ^*1^
BUN mg/dL	17.3 ± 2.2	115.0 ± 13.0 ^*1^	134.7 ± 5.3 ^*1^	130.5 ± 1.0 ^*2^
Crea mg/dL	0.4 ± 0.0	2.8 ± 0.3 ^*1^	4.1 ± 0.3 ^*1^	2.3 ± 0.2 ^*1,*3^
HCT %PCU	38.8 ± 0.5	28.0 ± 1.0 ^*1^	23.7 ± 2.0 ^*1^	29.8 ± 2.0 ^*1^
Hb g/dL	13.2 ± 0.2	9.6 ± 0.4 ^*1^	8.07 ± 0.7 ^*1^	10.1 ± 0.7 ^*1^
Serum Pi mg/dL	7.1 ± 0.56	22.2 ± 1.5 ^*1^	17.2 ± 0.9 ^*1^	25.5 ± 2.8 ^*1^
Body weight g	581.2 ± 24.5	262.6 ± 1.1 ^*1^	318.3 ± 12.1 ^*1^	242.2 ± 7.1 ^*1,*3^

The No-Fe group, PO-Fe group and IP-Fe group were CKD rats without Fe administration, with peroral Fe administration and with intraperitoneal Fe administration, respectively. Data are expressed as the mean ± SEM and were compared by one-way analysis of variance followed by the Tukey–Kramer test. A two-tailed *P* < 0.05 was considered statistically significant; *1; *p* < 0.05 vs. the control group, *2; *P* < 0.05 vs. the No-Fe group, *3; *p* < 0.05 vs. the PO-Fe group.

Abbreviations: iCa, ionized calcium; TCO_2_, total CO_2_; Glu, glucose; BUN, blood urea nitrogen; Crea, creatinine; HCT, hematocrit; Hb, hemoglobin; Pi, phosphorus.

### Iron and its related parameters in the liver

**Fig. 1:**
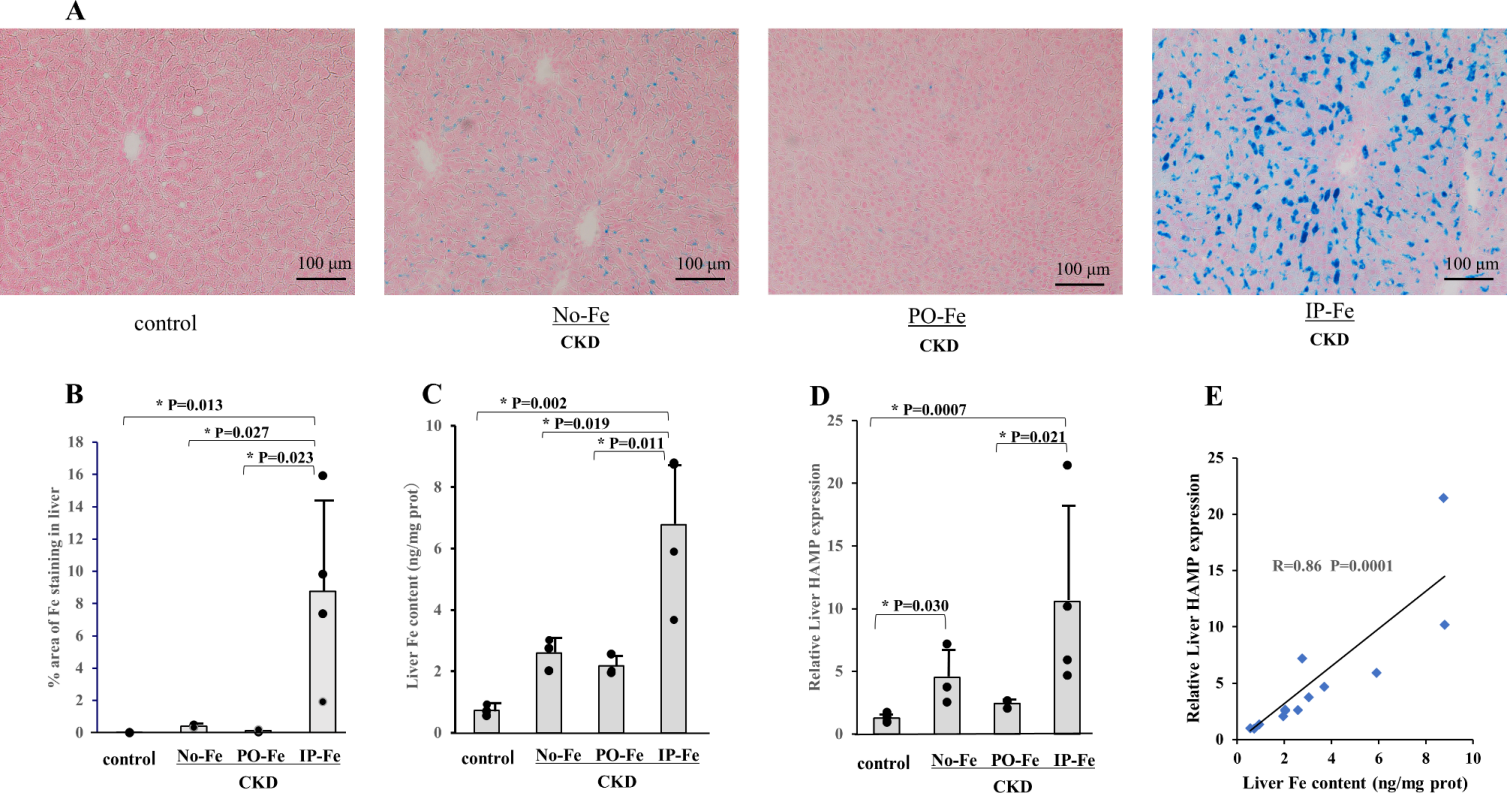
Effect of iron administration on liver Fe staining using Prussian blue (**A**), % area of Fe staining (**B**), Fe contents (**C**) and HAMP (hepcidin) expression (**D**) in the adenine-treated rats fed a high-phosphorus diet, and the correlation between the liver Fe contents and relative liver HAMP expression (R = 0.86, *p* = 0.0001) (**E**). The No-Fe group, PO-Fe group and IP-Fe group were CKD rats without Fe administration, with peroral Fe administration and with intraperitoneal Fe administration, respectively. Data are expressed as the mean ± SD and were compared by one-way analysis of variance followed by the Tukey–Kramer test. Linear regression analyses were used to determine the correlations between parameters. A two-tailed *p* < 0.05 was considered statistically significant

Berlin blue staining of the liver in the control group was not identifiable, while in the IP-Fe group, remarkable Fe deposits were observed (Fig. 1A). In the No-Fe and PO-Fe groups, iron was retained in the liver, albeit mildly. The liver % area of Fe staining and the Fe content were markedly higher in the IP-Fe group than in the other groups (Fig. 1B and C). Liver mRNA level of HAMP in the IP-Fe group showed a significant increase compared with that in the No-Fe and PO-Pe groups (Fig. 1D). HAMP expression in the No-Fe group was significantly higher than that in the control group, and there was no difference between the control and PO-Fe groups (Fig. 1D). In the analyses including all rats, relative liver HAMP expression was closely correlated with the liver Fe content (R = 0.83, *p* = 0.0001) (Fig. 1E).

### Iron content, relative ferritin H and Nrf2 expression in the aorta

**Fig. 2:**
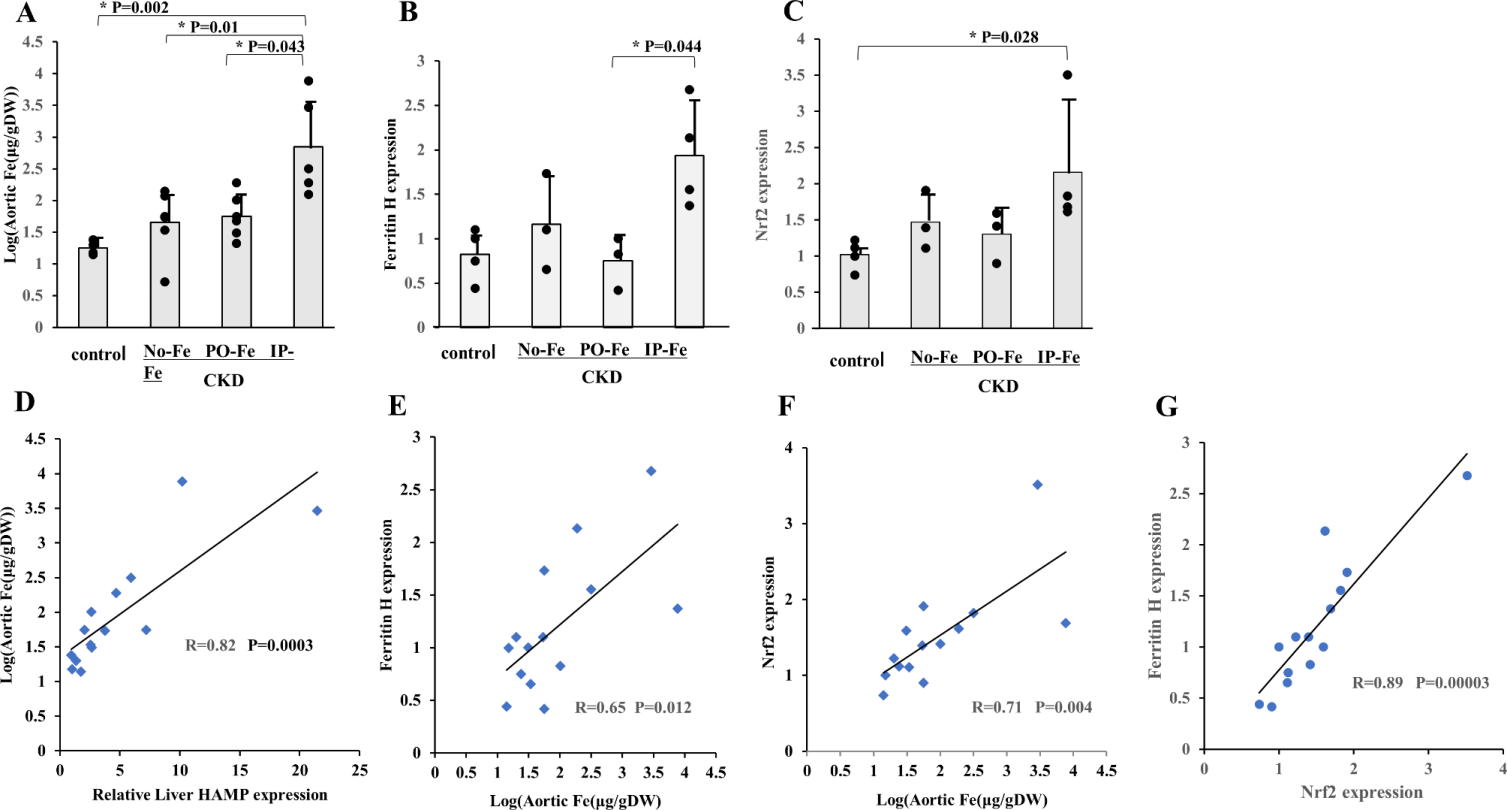
Effect of iron administration on the log-transformed Fe content (**A**), the relative mRNA levels of ferritin H (**B**) and Nrf2 (**C**) in the aortas of the adenine-treated rats fed a high-phosphorus diet, and the correlation between the relative liver mRNA level of HAMP and log-transformed aortic Fe content (R = 0.82, *p* = 0.0003) (**D**), between the log-transformed aortic Fe content and relative ferritin H expression in the aorta (R = 0.65, *p* = 0.012) (**E**), between the log-transformed aortic Fe content and the aortic mRNA level of Nrf2 (R = 0.71, *p* = 0.004) (**F**), and between the aortic mRNA level of Nrf2 and aortic mRNA level of ferritin H (R = 0.89, *p* = 0.00003) (**G**). The No-Fe group, PO-Fe group and IP-Fe group were CKD rats without Fe administration, with peroral Fe administration and with intraperitoneal Fe administration, respectively. Data are expressed as the mean ± SD and were compared by one-way analysis of variance followed by the Tukey–Kramer test. Linear regression analyses were used to determine the correlations between parameters. A two-tailed *p* < 0.05 was considered statistically significant

Aortic Fe was significantly higher in the IP-Fe group than in the other groups and was comparable among the control, No-Fe and PO-Fe groups (Fig. 2A). In the IP-Fe group, the mRNA level of ferritin H was significantly higher than that in the PO-Fe group (Fig. 2B). The mRNA level of Nrf2 in the IP-Fe group was significantly higher than that in the control group, but the expression in the No-Fe and PO-Fe groups was comparable to that in the control group (Fig. 2C). Intriguingly, we observed a significant relationship between liver mRNA level of HAMP and aortic Fe (R = 0.82, *p* = 0.0003) (Fig. 2D). In the analyses including all rats, the aortic mRNA level of ferritin H was associated with aortic Fe (R = 0.65, *p* = 0.012) (Fig. 2E). From the analyses of the association of mRNA level of Nrf2 with aortic Fe and mRNA level of ferritin H, aortic Fe (R = 0.71, *p* = 0.004) contents and ferritin H expression (R = 0.89, *p* = 0.00003) were associated with Nrf2 expression (Fig. 2F and G).

### Calcium (Ca) and phosphorus (Pi) contents and RUNX2 expression in the aorta

**Fig. 3:**
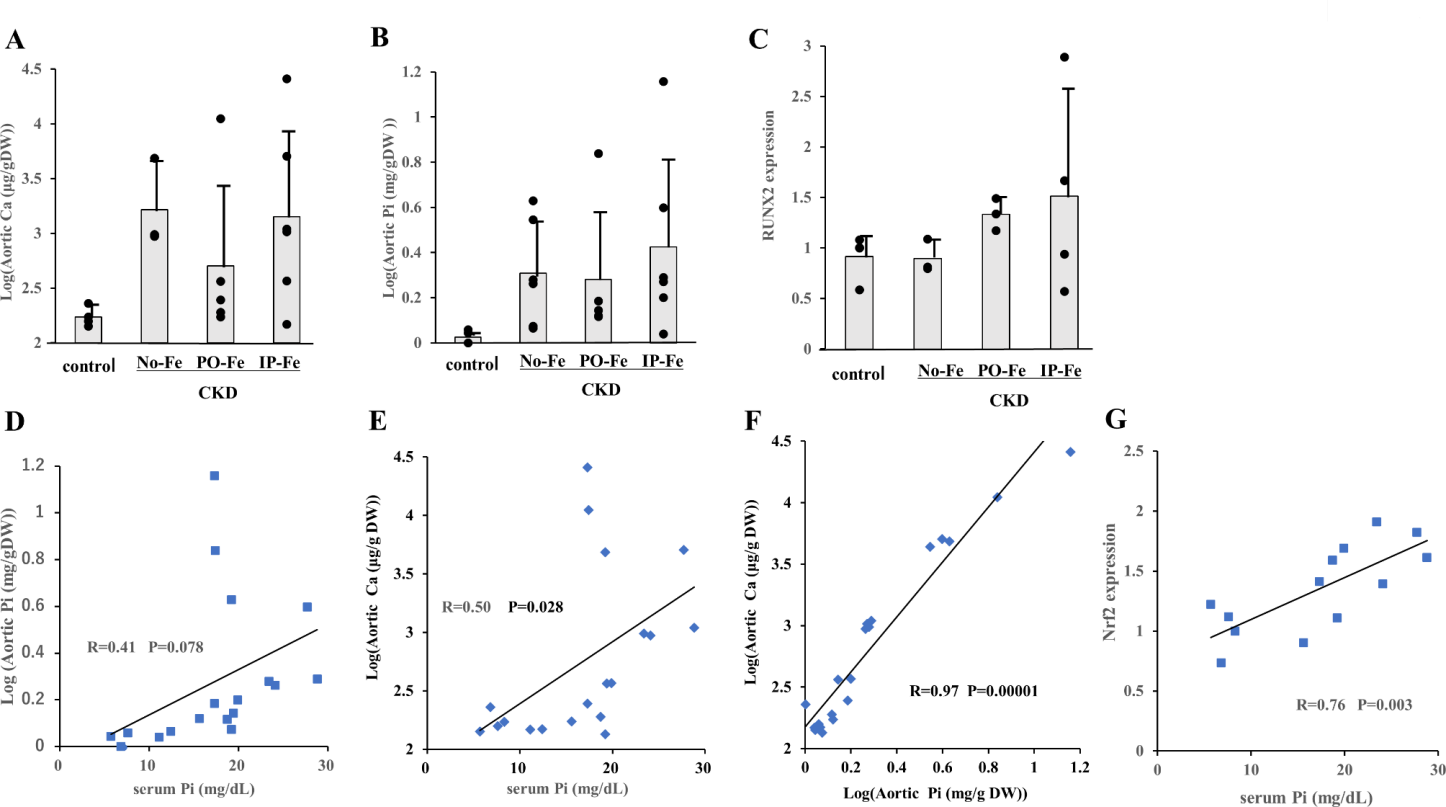
Effect of iron administration on the log-transformed Ca (**A**) and Pi (**B**) contents and relative mRNA level of RUNX2 (**C**) in the aortas of the adenine-treated CKD rats fed a high-phosphorus diet and the correlations between the serum Pi and log-transformed aortic Pi (R = 0.41, *p* = 0.078) (**D**) or Ca (R = 0.50, *p* = 0.028) (**E**) contents, between the log-transformed aortic Pi and Ca contents (R = 0.97, *p* = 0.00001) (**F**) and between the serum Pi and aortic mRNA level of Nrf2 (R = 0.76, *p* = 0.003) (**G**). The No-Fe group, PO-Fe group and IP-Fe group were CKD rats without Fe administration, with peroral Fe administration and with intraperitoneal Fe administration, respectively. Data are expressed as the mean ± SD and were compared by one-way analysis of variance followed by the Tukey–Kramer test. Linear regression analyses were used to determine the correlations between parameters. A two-tailed *p* < 0.05 was considered statistically significant. Abbreviations: Ca, calcium; Pi, phosphate: Fe, iron

To assess the effect of Fe administration on VC, we measured the Ca content in the aorta. Aortic Pi was also measured in all rats. Neither the aortic Ca nor the aortic Pi content was significantly different among the groups (Fig. 3A and B). The aortic mRNA level of RUNX2 was comparable in all groups (Fig. 3C). However, aortic Ca was higher in all CKD groups, that is, the No-Fe, PO-Fe, and IP-Fe groups, than in the control group (*p* = 0.0011). Thus, whether the Fe was administered orally or parenterally (intraperitoneal injection), it was unlikely to affect the aortic Ca content. Serum Pi levels were significantly related to aortic Ca levels (R = 0.5, *p* = 0.028) but not to aortic Pi levels (R = 0.41, *p* = 0.078) (Fig. 3D and E). The aortic Ca content was closely correlated with the aortic Pi content (R = 0.97, *p* = 1 × 10^− 12^) (Fig. 3F). From the analyses of the association of mRNA level of Nrf2 with serum Pi, the serum Pi were associated with Nrf2 expression significantly (R = 0.76, *p* = 0.003) (Fig. 3G). There was no apparent relationship between the aortic Ca and Fe levels,

### Aortic mRNA expression levels of HIF-1α, HMOX1, and Nqol1

**Fig. 4:**
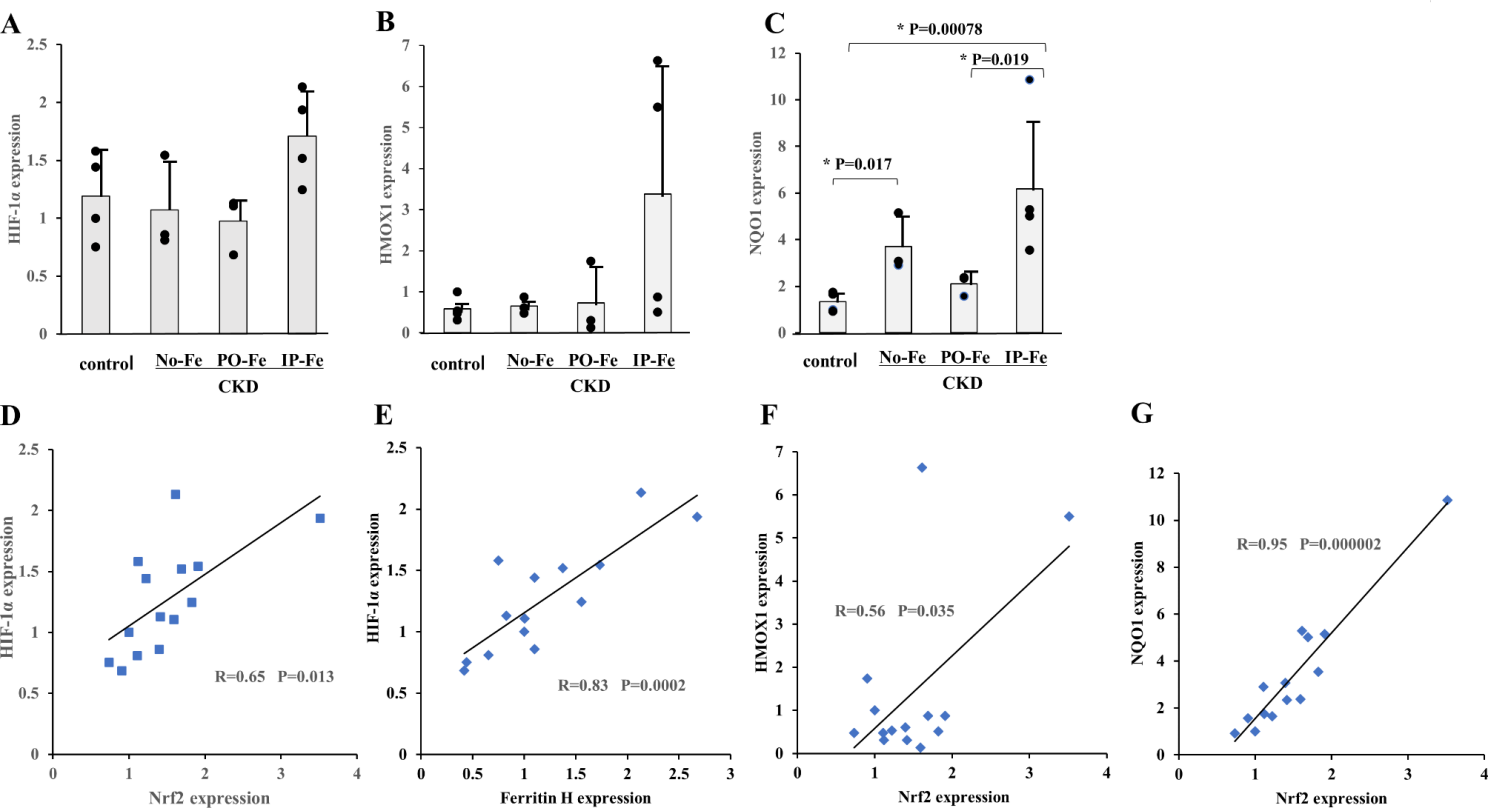
Effect of iron administration on the relative mRNA levels of HIF-1α (**A**), Heme Oxygenase 1 (HMOX1) (**B**), and NAD(P)H quinone dehydrogenase 1 (NQO1) (**C**) in the adenine-treated rats fed a high-phosphate diet and the correlations between aortic Nrf2 mRNA levels and HIF-1α mRNA levels (R = 0.65, *p* = 0.013) (**D**), between aortic ferritin H mRNA levels and HIF-1α mRNA levels (R = 0.83, *p* = 0.0002) (**E**), between aortic Nrf2 mRNA levels and HMOX1 mRNA levels (R = 0.56, *P* = 0.035) (**F**), and between aortic Nrf2 mRNA levels and NQO1 mRNA levels (R = 0.95, *P* = 0.000002) (**G**). The No-Fe group, PO-Fe group and IP-Fe group were CKD rats without Fe administration, with peroral Fe administration and with intraperitoneal Fe administration, respectively. Data are expressed as the mean ± SD and were compared by one-way analysis of variance followed by the Tukey–Kramer test. Linear regression analyses were used to determine the correlations between parameters. A two-tailed *p* < 0.05 was considered statistically significant

The mRNA level of HIF-1α was comparable in all groups (Fig. 4A). The mRNA level of HIF-1αwas associated with that of Nrf2 (R = 0.65, *p* = 0.013) (Fig. 4D). From the analysis of the association of the aortic mRNA level of HIF-1α with that of ferritin H, they were significantly correlated (R = 0.83, *p* = 0.0002) (Fig. 4E).

Because we could not confirm the expression of Nrf2 protein in this study, we examined the expression of genes positively regulated by Nrf2 protein, such as HMOX1 and NQO1. The aortic mRNA level of HMOX1 was comparable in all groups but significantly correlated with the aortic mRNA level of Nrf2 (R = 0.56, *p* = 0.035) (Fig. 4B and F). The aortic mRNA level of NQO1 was significantly higher in the IP-Fe group than those in the control and PO-Fe groups and was closely correlated with the aortic mRNA level of Nrf2 (R = 0.95, *p* = 0.000002) (Fig. 4C and G). Therefore, we can assume that Nrf2 protein was increased along with its mRNA.

There were no apparent relationships between the aortic Ca content and the mRNA levels of Nrf2, ferritin H, HIF-1α and RUNX2, and between mRNA levels of HIF-1α and RUNX2 (data not shown).

## Discussion

### Iron administration, liver iron and aortic iron deposition

In the present study, in a rat model of CKD fed a high-Pi diet, parenteral iron administration, but not the other methods of iron administration, caused a significant increase in the liver Fe content and stainable Fe deposits (Fig. 1A, B and C). Correspondingly, liver HAMP expression was the highest in the IP-Fe group (Fig. 1D). Strikingly, a very close correlation was observed between HAMP mRNA expression in the liver and the Fe content in the aorta (Fig. 2D). Hepcidin inhibits the function of FPN to pump iron out of the cell and causes iron sequestration into the cell. It is not known which cellular sites in the aorta predominantly accumulate iron. The aorta has a complex three-layered structure mainly consisting of endothelial cells, smooth muscle cells, and connective tissues: the inner layer (intima), the middle layer (tunica media), and the outer layer (adventitia). The presence of FPN in endothelial cells has been confirmed, albeit in cultured cell models such as human umbilical endothelial cells (HUVECs) [28], human coronary artery endothelial cells [29] and human pulmonary artery endothelial cells [30], while the presence of FPN in VSMCs has been confirmed in pulmonary artery smooth muscle cells (PASMCs) [31, 32]. From these observations, we may assume that hepcidin could sequester iron in aortic tissues, including endothelial cells and smooth muscle cells.

Notably, no difference in liver Fe levels was observed between the No-Fe and PO-Fe groups. These observations could be consistent with a previous study that reported that an increase in the Fe concentration of the diet by adding ferrous sulfate only marginally influenced liver Fe levels [33]. We suspected that “mucosal block”, which is characterized by decreased uptake after the administration of excess iron, might affect enteric Fe absorption, as hepatic HAMP expression was not increased in the PO-Fe group [34–36].

### Effect of Fe administration on the aortic Ca and Pi contents

In the present study, we tested the effect of Fe administration on VC or the aortic Ca content, and no effect on VC was observed with either oral or parenteral Fe administration, while in previous studies, conflicting results have been obtained [14, 15, 17]. We suspect that this may be due not only to the variability among individual animals but also to the amount and type of Fe used. Regarding the effect of the Fe dosage on VC in the adenine-treated phosphorus loading model, VC was suppressed in rats treated with 40 mg/week iron dextran for 5 weeks [14] and mice treated with 200 mg/kg iron dextran three times every other day [15], while it increased in a non-CKD rat study with 250 mg/kg iron dextran 5 days a week for 4 weeks [17]. Thus, it is presumed that higher doses of Fe administration may promote VC, while suppressive use may attenuate VC.

In addition to the effect of the amount of Fe, the type of Fe preparation used may also affect VC. We used saccharated ferric oxide (SFO) in this study because this Fe preparation is predominantly used in clinical practice in Japan. We observed that in the IP-Fe group, SFO administration caused a significant reduction in blood iCa levels, which may attenuate aortic Ca deposition (Table 2). Similarly, in a previous report, SFO-treated healthy rats showed a decline in iCa, whereas those treated with iron dextran did not [37]. Further investigations are needed to clarify the effects of the Fe dosage and type of Fe preparation on VC.

### Activation of Nrf2-induced genes encoding antioxidative proteins with mutually opposing effects on vascular calcification: ferritin H and HIF-1α

In the present whole animal study, aortic Nrf2 mRNA levels were significantly higher in the IP-Fe group than in the other groups and were closely related to aortic Fe levels and serum phosphate, which has not been demonstrated in previous studies. We may presume that oxidative stress due to aortic increases in Fe and Pi might accelerate Nrf2 expression. Previous studies using VSMCs have established that Nrf2 plays a central role in protecting cells from oxidative stress and attenuates VC [20, 21, 38]. However, no correlation was found between Nrf2 expression and aortic Ca levels, which may indicate that Nrf2 and its inducible proteins inhibit oxidative stress but do not necessarily attenuate VC.

Gene transcription of Nrf2-related antioxidant proteins is induced via antioxidant response elements (AREs) [39]. In the present study, we demonstrated significant correlations between the mRNA levels Nrf2 and ferritin H, as well as between Nrf2 and HIF-1α (Figs. 2G and 4D).

Ferritin H, an iron-containing molecule, has been demonstrated to be involved in the inhibitory mechanism of Pi-mediated osteoblastic differentiation of human smooth muscle cells and VC, as it has ferroxidase activity and antioxidant properties [22, 23]. In the present study, aortic ferritin H mRNA levels were significantly higher in the IP-Fe group than in the PO-Fe group and were closely related to aortic Fe levels (Fig. 2B and E), as demonstrated in previous studies showing that ferritin transcription and translation are precisely regulated by intracellular Fe levels [40]. Although ferritin H may have a protective effect against VC, ferritin H and the aortic Fe content were not linked with the aortic Ca content in the present study.

Furthermore, the HIF-1α promoter was found to contain an ARE, the putative Nrf2 binding site [24]. HIF-1α indeed reduces ROS production by multiple mechanisms, including the modulation of mitochondrial ROS production over the physiological range of O_2_ [41, 42]. However, in cultured VSMCs, HIF-1α activation alone has been reported to increase intracellular Ca levels [26]. Additionally, high Pi conditions could block the interaction between hydroxylated HIF-1α by prolyl-4-hydroxylase domain (PHD) proteins and von Hippel‒Lindau protein for polyubiquitylation [26]. Thus, HIF-1α degradation was repressed by high Pi conditions, leading to increased HIF-1α stabilization, even under normal oxygenation [25, 26]. Therefore, hyperphosphatemia may promote VC not only through activation of HIF-1α at the genetic level via Nrf2 but also through stabilization of HIF-1α at the protein level.

VC may occur based on the balance of two antagonistic factors, namely, ferritin H and HIF-1α. This phenomenon might be an appropriate explanation for the contradiction between the presumption that increased Nrf2 expression by higher serum phosphorus levels could attenuate oxidative stress and suppress VC and the observation that elevated Pi steadily promoted VC.

As NRF2 and HIF-1α proteins are constitutively expressed by cells, both are rapidly degraded under homeostatic/normoxic conditions via the NRF2-Kelch-like ECH-associated protein l (KEAP1) axis or prolyl hydroxylase domain enzymes [43]. In the present study, however, we did not examine the expression of HIF-1α and ferritin H at the protein level. Furthermore, there are many more factors to be studied; that is, antioxidative proteins induced by Nrf2, other than HIF-1α and ferritin H, may also affect VC. Adding complexity to the mechanistic understanding of VC is that oxidative stress directly promotes HIF-1α expression via NF-kB [44]. Further studies are needed to clarify whether antagonism of ferritin H and HIF-1α is an important factor in determining the mechanisms for the progression of VC.

## Conclusion

Parenteral Fe administration markedly increased liver HAMP expression and the aortic Fe content, which might be related to the inhibition of iron egress from aortic tissues. Neither route of iron administration significantly affected the aortic Ca contents. As there were reciprocal correlations among the serum Pi, aortic Pi and Ca contents, we confirmed that the pathological flow of VC that elevated serum Pi and increased aortic Pi was closely related to the aortic Ca content. Both parenteral iron administration and the increase in serum Pi concentration appeared to enhance the expression of Nrf2 in the aorta. From these observations, we suspected that oxidative stress due to aortic increases in the Fe content and high phosphate might accelerate Nrf2 expression. Aortic Nrf2 expression was related to the expression of its induced antioxidative proteins ferritin H and HIF-1α. Thus, Nrf2 expression might induce the expression of ferritin H, which suppresses VC, as well as HiF-1α, which promotes VC, and their opposing effects on VC may complicate the elucidation of the pathogenesis of VC.

## Data Availability

The datasets used and/or analyzed during the current study are available from the corresponding author on reasonable request.
